# Draft Genome Sequence of *Aspergillus persii* NIBRFGC000004109, Which Has Antibacterial Activity against Plant-Pathogenic Bacteria

**DOI:** 10.1128/genomeA.00932-17

**Published:** 2017-09-21

**Authors:** Jung A. Kim, Jongbum Jeon, Sook-Young Park, Ki-Tae Kim, Gobong Choi, Hoa Thi Nguyen, Sun Jeong Jeon, Hyang Burm Lee, Chang-Hwan Bae, Hee-sun Yang, Joo-Hong Yeo, Jin-Cheol Kim, Yong-Hwan Lee, Soonok Kim

**Affiliations:** a Genetic Resources Assessment Division, National Institute of Biological Resources, Incheon, South Korea; b Department of Agricultural Biotechnology, Interdisciplinary Program in Agricultural Genomics, Center for Fungal Genetic Resources, and Center for Fungal Pathogenesis, Seoul National University, Seoul, South Korea; c Department of Plant Medicine, Sunchon National University, Suncheon, South Korea; d Department of Agricultural Chemistry, Chonnam National University, Gwangju, South Korea

## Abstract

The fungus *Aspergillus persii* strain NIBRFGC000004109 is capable of producing penicillic acid and showed antibacterial activity against various plant-pathogenic bacteria, including *Xanthomonas arboricola* pv. pruni. Here, we report the first draft whole-genome sequence of *A. persii*. The assembly comprises 38,414,373 bp, with 12 scaffolds.

## GENOME ANNOUNCEMENT

Bioactive compounds from fungi provide an important resource as a lead molecule for the development of novel chemicals. *Aspergillus* spp. are promising fungi for the production of various useful chemicals. We recently found that *Aspergillus persii* strain NIBRFGC000004109 showed strong antibacterial activity, with penicillic acid as an active compound, especially against plant-pathogenic bacteria, including *Agrobacterium tumefaciens*, *Ralstonia solanacearum*, *Xanthomonas arboricola* pv. pruni, and *Xanthomonas oryzae* pv. oryzae (1). The genome sequence of *A. persii* will help us understand the genetic machinery of penicillic acid biosynthesis. Here, we report the first draft genome sequence of *Aspergillus persii* NIBRFGC000004109, which was isolated from barley grain (*Hordeum vulgare*) harvested in 2012 (1).

Genomic DNA was extracted from germlings grown in yeast extract broth by shaking at 150 rpm overnight at 23°C, using the DNeasy minikit (Qiagen, Valencia, CA, USA). A total of 24.62 Gb of genome sequences were produced using 1 paired-end and 2 mate pair libraries on HiSeq 2000 (Illumina) and 8 cells on PacBio RSII platforms at the Theragen Etex Bio Institute (Suwon, South Korea), representing 616.57-fold coverage. Reads containing the nuclear genome were assembled into 12 scaffolds (501 contigs), with an *N*_50_ value of 4.60 Mb, using a mixed pipeline of FALCON, an assembler of PacBio reads, and SOAP*denovo* version 2, an assembler of Illumina reads (2). The respective assembled reads were merged with HaploMerger2 (3). The total length of the assembled genome was 38,414,373 bp, with a G+C content of 48.64%.

A total of 12,328 protein-coding genes were identified after gene prediction and annotation based on the AUGUSTUS software (4). Using the previously developed gene family pipelines (5–10), we found 673 transcription factor genes, 159 cytochrome P450 genes, 1,079 genes encoding secretory proteins, 61 genes encoding plant cell wall-degrading enzymes, 8 genes encoding laccases, and 33 genes encoding peroxidases. Additionally, 68 polyketide synthase and 77 nonribosomal peptide synthetase genes were identified using the antiSMASH software (11, 12). This is the first report of the genome sequence of *A. persii* and its analysis. This draft genome sequence will provide valuable information to identify genes for the biosynthesis of penicillic acid and will also serve as a platform to facilitate comparative genomics with other *Aspergillus* fungi for evolutionary biology.

### Accession number(s).

The draft genome sequence of *Aspergillus persii* NIBRFGC000004109 has been deposited in GenBank under accession no. NGZO00000000. The version described in this article is the first version, NGZO01000000.
